# Clinical, genetic aspects and molecular pathogenesis of osteopetrosis

**DOI:** 10.18699/VJGB-23-46

**Published:** 2023-07

**Authors:** D.D. Nadyrshina, R.I. Khusainova

**Affiliations:** Ufa University of Science and Technology, Ufa, Russia; Ufa University of Science and Technology, Ufa, Russia Saint Petersburg State University, St. Petersburg, Russia

**Keywords:** osteopetrosis, classification, connective tissue, остеопетроз, классификация, соединительная ткань

## Abstract

Osteopetrosis (“marble bone”, ICD-10-78.2) includes a group of hereditary bone disorders distinguished by clinical variability and genetic heterogeneity. The name “osteopetrosis” comes from the Greek language: ‘osteo’ means ‘bone’ and ‘petrosis’ means ‘stone’, which characterizes the main feature of the disease: increased bone density caused by imbalances in bone formation and remodeling, leading to structural changes in bone tissue, predisposition to fractures, skeletal deformities. These defects, in turn, affect other important organs and tissues, especially bone marrow and the nervous system. The disease can be autosomal recessive, autosomal dominant, X-linked or sporadic. Autosomal dominant osteopetrosis has an incidence of 1 in 20,000 newborns and autosomal recessive one has 1 in 250,000. To date, 23 genes have been described, structural changes in which lead to the development of osteopetrosis. Clinical symptoms in osteopetrosis vary greatly in their presentation and severity. The mildest skeletal abnormalities are observed in adulthood and occur in the autosomal dominant form of osteopetrosis. Severe forms, being autosomal recessive and manifesting in early childhood, are characterized by fractures, mental retardation, skin lesions, immune system disorders, renal tubular acidosis. Clinical examination and review of radiographs, bone biopsy and genetic testing provide the bases for clinical diagnosis. The early and accurate detection and treatment of the disease are important to prevent hematologic abnormalities and disease progression to irreversible neurologic consequences. Most patients die within the first decade due to secondary infections, bone marrow suppression and/or bleeding. This article summarizes the current state of the art in this field, including clinical and genetic aspects, and the molecular pathogenesis of the osteopetrosis.

## Abstract (ru)

Остеопетроз («мраморная кость», МКБ-10-78.2) включает группу наследственных нарушений костной системы, отличающихся клинической вариабельностью и генетической гетерогенностью. Название «остеопетроз» происходит от греческих слов: ỏστέον (остео) – кость, πέτρα (петросис) – камень, что характеризует основной признак заболевания – повышенную плотность костей, обусловленную нарушениями равновесия формирования и ремоделирования кости, приводящими к структурным изменениям в костной ткани, предрасположенности к переломам, деформациям скелета. Эти дефекты, в свою очередь, влияют на другие важные органы и ткани, особенно на костный мозг и нервную систему. Заболевание наследуется по аутосомно-рецессивному, аутосомно-доминантному типам, встречаются Х-сцепленные формы заболевания, а также спорадические случаи. Частота аутосомно-доминантного остеопетроза составляет 1 на 20 тыс., а аутосомно-рецессивного – 1 на 250 тыс. новорожденных. На сегодняшний день описано 23 гена, структурные изменения в которых приводят к развитию остеопетроза. Клинические симптомы при остеопетрозных состояниях сильно различаются по проявлению и степени тяжести. Наиболее легкие скелетные нарушения наблюдаются во взрослом возрасте и встречаются при аутосомно-доминантной форме остеопетроза. Тяжелые формы, характеризующиеся переломами, умственной отсталостью, поражениями кожи, нарушениями иммунной системы, ацидозом почечных канальцев, наблюдаются при аутосомно-рецессивном типе остеопетроза, проявляющемся в раннем детском возрасте. Диагноз «остеопетроз» ставится на основании клинической и рентгенологической оценки, подтвержденной биопсией костей и генетическим тестированием. Для аутосомно-рецессивных форм остеопетроза важны ранняя диагностика и лечение заболевания с целью установления гематологических нарушений, а также для предотвращения прогрессирования заболевания до возникновения необратимых неврологических последствий. Большинство пациентов умирают в течение первого десятилетия из-за вторичных инфекций и/или кровотечений, а также угнетения функции костного мозга. В настоящей работе представлен обзор современного состояния изучения остеопетроза, клинико- генетических аспектов, молекулярного патогенеза заболевания

## Introduction

Bone is a dynamic tissue that undergoes constant selfrenewal; bone tissue homeostasis depends on the functional balance between three cell types: osteoclasts necessary for bone resorption; osteoblasts responsible for bone matrix formation, and osteocytes involved in the reception and transduction of mechanical stimuli and in the regulation of osteoclast/osteoblast differentiation and function. The balance between bone synthesis and resorption is finely tuned and any perturbations of this balance in adults trigger bone disease (Coudert et al., 2015).

Osteopetrosis is a group of inherited metabolic bone diseases characterized by increased bone mass due to defects in osteoclast function or formation, leading to fractures, generalized osteosclerosis, pancytopenia, and in severe cases, cranial neuropathies and hepatosplenomegaly. Abnormalities in the structural organization of multiple genes are responsible for the development of the disease, leading to marked clinical heterogeneity

## Classification and clinical features of osteopetrotic conditions

In 2006, the Nosology Group of the International Skeletal Dysplasia Society presented a classification of increased bone density conditions into several distinct entities based on clinical features, mode of inheritance and underlying molecular and pathogenetic mechanisms (Stark, Savarirayan, 2009). 13 clinical forms of osteopetrosis were identified: severe neonatal or infantile forms of osteopetrosis, the intermediate form of osteopetrosis with renal tubular acidosis, the late form (Albers-Schönberg disease), osteopetrosis with ectodermal dysplasia and immune defect (OLEDAID), leukocyte adhesion deficiency syndrome (LAD-III) and osteopetrosis, pycnodisostosis, osteopoikilosis, melorheostosis with osteopoikilosis, dysosteosclerosis, osteomesopiknosis, congenital striated osteopathy with cranial stenosis, Stanescu-type osteosclerosis (Stark, Savarirayan, 2009).

Recently, the advent of next-generation sequencing technology has continued to identify new molecular causes of the disease, leading to an expansion of the classification. We have systematized all currently known osteopetrosis conditions with a description of the genetic defect and the main clinical characteristics, and showed it in Table.

**Table 1.**
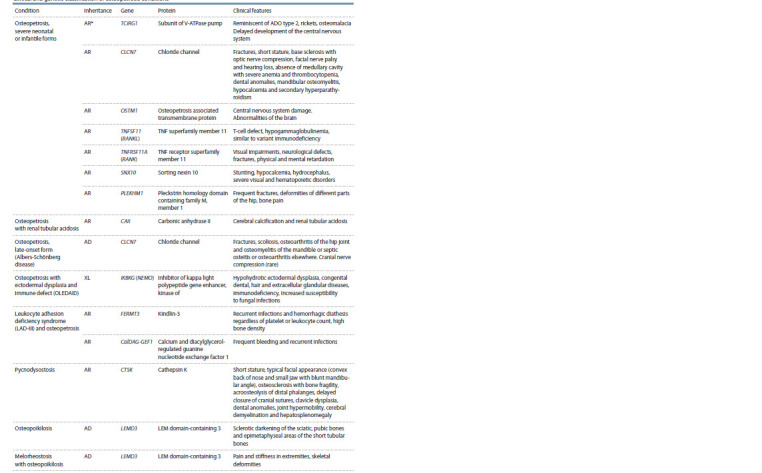
Clinical and genetic classification of osteopetrosis conditions

**Table 1end.**
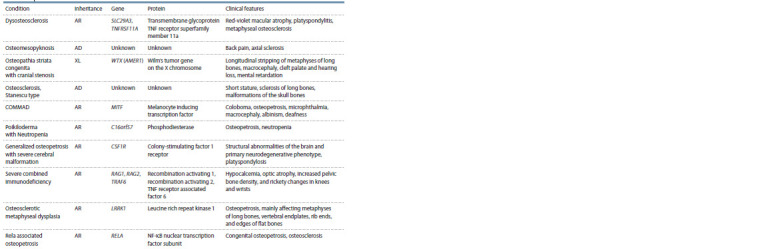
Table 1end * Mode of inheritance: AD – autosomal-dominant, AR – autosomal-recessive.

Autosomal recessive, intermediate forms of osteopetrosis, and autosomal dominant and X-linked type of osteopetrosis have been described

Autosomal dominant osteopetrosis (Albers-Schönberg disease, ADO) is commonly referred to as benign osteopetrosis, with an incidence of 1 : 20,000 newborns. Clinical and radiologic signs of ADO most often appear late in childhood or adolescence. The major complications are skeletal, including fractures, scoliosis, osteoarthritis of the hip joint, and osteomyelitis, especially affecting the mandible in combination with dental abscesses or dental caries. About 70 % of patients with ADO have mutations in the CLCN7 gene. In the remaining ~30 % of cases, no mutations in the CLCN7 gene sequences were found, suggesting involvement of additional genes in the pathogenesis of this form of osteopetrosis (Coudert et al., 2015).

The X-linked form of osteopetrosis results from mutations in the IKBKG gene. Patients with this type have a specific phenotype with ectodermal dysplasia and an immune defect

Intermediate forms of osteopetrosis are caused by defects in the PLEKHM1 and SNX10 genes. Clinical manifestations vary in patients with this type.

The most severe malignant disease states are autosomal recessive forms of osteopetrosis (ARO), which are caused by defects in various genes with products that are involved in the formation, function and differentiation of osteoclasts. Clinically, patients with ARO are characterized by severe disorders of the musculoskeletal system, central nervous system (CNS), manifesting in the first few months of life. Patients are treated in pediatric or hematologic departments. Sick children have recurrent infections. They also suffer from frequent bleeding secondary to medullary hyperplasia caused by bone invasion of the medullary space. Cranial nerve compression can lead to blindness and deafness. Neurologic defects may also occur in some patients regardless of nerve compression. Radiological examination reveals dense bones characterized by extreme fragility.

Currently, there is no universal therapy regimen for osteopetrosis and strategies and tactics in the treatment of the disease are determined by its molecular pathogenesis, so it is necessary to identify the genetic cause of the disease in each individual case. Hematopoietic stem cell transplantation (HSCT) is recognized as the most effective treatment, which allows restoration of bone resorption by cells of donor origin. This therapy is suitable for patients with mutations in the TCIRG1, TNFRSF11A (RANK), SNX10, CAII, IKBKG, FERMT3, CalDAG-GEF1 genes. Patients with severe neurological disorders caused by mutations in the TNFSF11 and OSTM1 genes are not indicated for HSCT and only symptomatic treatment is available for these patients. Risky and invasive transplant procedures are also not indicated for mild forms of the disease, for example, with mutations in the PLEKHM1, SLC29A3, CTSK, or CLCN7 genes. For recently diagnosed forms of the disease, there is currently insufficient knowledge to determine specific treatment tactics

Thus, osteopetrosis is a clinically variable disease with a wide spectrum of clinical manifestations and symptoms of varying severity. It is necessary to understand the molecular pathogenesis of the disease in order to correctly diagnose and determine the treatment tactics of the disease.

##  Molecular pathogenesis of different forms of osteopetrosis

The disease is characterized by a complex molecular pathogenesis caused by mutations in 23 genes (see the Table) responsible for the development of corresponding clinical osteopetrosis conditions (TCIRG1, CLCN7, OSTM1, PLEKHM1, SNX10, TNFSF11 (RANKL), TNFRSF11A (RANK ), IKBKG (NEMO), RAG1, RAG2, TRAF6, FERMT3, LRRK1, MITF, C16orf57, CSF1R, CAII, SLC29A3, CalDAG-GEF1, CTSK, WTX, LEMD3, RELA).

Autosomal recessive forms of osteopetrosis arise from mutations in genes that are involved in osteoclast function (osteoclast-rich) or differentiation (osteoclast-poor forms of osteopetrosis).

Osteoclast-rich osteopetrosis is caused by mutations in genes responsible for lacunar acidification, resorption and pH regulation (TCIRG1, CLCN7, OSTM1 and CAII ), vesicular transport and sorting of protein complexes to the membrane (SNX10 and PLEKHM1), lysosomal nucleoside transport (SLC29A3) cytoskeletal rearrangement for “corrugated edge” formation (KINDLIN3, integrin-β and LRRK1) and lysosomal proteolytic cleavage for bone remodeling and resorption (CTSK), for signal transduction and osteoclast function (MITF, TRAF6, RELA and NEMO) (De Cuyper et al., 2021; Penna et al., 2021).

In osteopetrosis with osteoclast deficiency, osteoclast differentiation is impaired due to mutations in the TNFSF11 and TNFRSF11A genes encoding RANKL and its receptor RANK, respectively, or in the CSF1R gene encoding M- CSF. As a consequence, osteoclast precursors are unable to fuse and differentiate into multinucleated resorbing osteoclasts

About 50 % of patients with ARO have mutations in the TCIRG1 (T-cell immunoregulator 1) gene. This gene encodes a subunit of a large protein complex known as vacuolar H+-ATPase (V-ATPase), mainly expressed by osteoclasts and gastric parietal cells on apical membrane. The protein complex acts as a pump to move protons across the membrane. The V-ATPase pump acidifies the resorption lacuna in the bone for the dissolution of the hydroxyapatite crystals that form the bone mineral fraction and the degradation of the matrix

The a3 V-ATPase subunit is also involved in the interaction between the actin cytoskeleton and microtubules, necessary for the osteoclast ruffled border formation (corrugated paper). Accordingly, TCIRG1-mutated osteoclasts show defective ruffled border and markedly reduced resorptive activity. In addition, V-ATPase maintains low pH in the stomach for the dietary Ca2+ absorption, and because gastric acidification is also relevant for calcium uptake, this form of osteopetrosis is characterized by rickets or osteomalacia (Penna et al., 2019).

To date, more than 120 different mutations in the TCIRG1 gene have been described, including missense mutations, nonsense mutations, small insertions/deletions, large genomic deletions, and splicing defects, demonstrating the high genetic heterogeneity of the TCIRG1-deficient ARO cohort (Palagano et al., 2018).

Mutations in the CLCN7 (chloride potential-dependent channel 7) gene are responsible for 17 % of autosomal recessive osteopetrosis cases and for the majority of autosomal dominant osteopetrosis cases (70 %) (Penna et al., 2021). Bi-allelic mutations cause a very severe form of the disease in which bone defects and hematologic failure are combined in some patients with primary neurodegeneration resembling lysosomal accumulation disease, cerebral atrophy, spasticity, axial hypotonia, and peripheral hypertension. Conversely, single-allelic CLCN7 mutations result in autosomal dominant osteopetrosis and are associated with milder symptoms and later onset.

The CLCN7 gene encodes 2Cl–/H+-antiporters, regulated by a potential-dependent mechanism, expressed on the “corrugated edges” of osteoclasts and on the membranes of late endosomes and lysosomes. CLC family proteins transport chlorine ions across cell membranes to maintain membrane potential, regulate transepithelial Cl– transport, and control intravesical pH between different organelles.

The neuropathic form of autosomal recessive osteopetrosis is caused by mutations in the OSTM1 or CLCN7 genes. Mutations in OSTM1 (transmembrane protein 1 associated with osteopetrosis) account for about 5 % of ARO cases and invariably cause osteopetrosis and severe primary neurodegeneration with a life expectancy of less than two years. OSTM1 acts as the auxiliary β-subunit of CLC-7 to support bone resorption and lysosomal function.

Virtually all of the identified mutations in this gene result in protein shortening. A secreted form of shortened OSTM1 has been shown to inhibit osteoclast formation in vitro through suppression of the BLIMP1-NFATc1 axis, thereby providing a putative additional pathogenetic mechanism of OSTM1-deficient ARO. Moreover, using a specially designed quantitative PCR strategy, two different homozygous microdeletions spanning ~110 and ~10 bp, respectively, and affecting the N-terminal part of the OSTM1 gene were detected in two unrelated families of Arab and Indian origin consisting of five critically ill patients. Sequence analysis of the relevant genomic region identified AluSx-mediated recombination and nonrecurrent rearrangement followed by nonhomologous end joining as the respective underlying molecular mechanism (Palagano et al., 2018; Zhang et al., 2020).

Osteopetrosis with early onset neurodegeneration and iron accumulation in certain brain regions has been described in one patient, which is a very unusual finding. Full-exome sequencing revealed the presence of a novel c.783+5G>T mutation in the OSTM1 gene, causing exon 4 skipping, and a frameshift variant c.446dup in the homozygous state in the MANEAL gene. This gene encodes an endo-alpha-like mannosidase protein, which probably localizes in the Golgi complex and is potentially involved in glycoprotein metabolism; indeed, increased mannose tetrasaccharide molecules have been found in the patient’s urine and cerebrospinal fluid. How this might be related to iron accumulation in the brain and the contribution of a mutation in the MANEAL gene to the formation of the osteopetrosis phenotype requires further investigation.

Osteopetrosis with renal tubular acidosis and cerebral calcinosis is caused by mutations in the CAII gene. Carboanhydrase (CAII) is a zinc-containing metalloenzyme responsible for catalyzing the reversible conversion of carbon dioxide (CO2) and water (H2O) to bicarbonate (HCO3–) and protons (H+). Carboanhydrase helps in the maintenance of homeostasis in the body. The substrates and products of the reaction (CO2, HCO3– and H+) are necessary for the regulation of biological processes such as respiration, cerebrospinal fluid formation, and bone resorption (Sanyanga et al., 2019).

About 30 different mutations have been identified in the CAII gene: missense mutations, nonsense mutations, and splice site mutations. The majority of patients with this mutation are of Arabian origin

Intermediate forms of ARO caused by mutations in the PLEKHM1 (member 1 of the M family containing the pleckstrin homology domain) and SNX10 (sorting nexin 10) genes have been described (Coudert et al., 2015). The PLEKHM1 gene encodes a cytosolic protein involved in endosome transport pathways through interaction with small GTPases RAB7 and ARL8. In addition, PLEKHM1 is involved in the fusion of autophagosomes and lysosomes required for the clearance of a variety of protein aggregates. Accordingly, disruption of specific domains of this protein or its loss impairs vesicle distribution, secretion, and formation of corrugated wukras, thereby undermining the resorptive function of osteoclasts. PLEKHM1 is a large protein containing various functional domains: the RUN domain in which the c.296+1G>A mutation was localized, originally identified in two siblings with ARO; two plectrin homology (PH) domains separated by an LC3-interacting region (LIR); the Rubicon homology (RH) domain and the C1 zinc finger at the C-terminal.

Two different presumably dominant mutations in the PLEKHM1 gene have been reported in two unrelated patients: c.2140C>T (p.Arg714Cys), clearly unrelated to osteopetrosis, was found in the second PH domain; and the recently discovered c.3051_3052delCA mutation, located in the RH domain, is predicted to eliminate the zinc finger motif. The RH domain is essential for the interaction of PLEKHM1 with RAB7, leading to reduced interaction of the mutant protein with RAB7, resulting in abnormal intracellular localization and increased autophagy.

Less than 5 % of ARO cases are caused by mutations in the SNX10 (sorting nexin 10) gene, which encodes a protein family of cytoplasmic and membrane-bound proteins characterized by a phosphoinositide-binding domain called the PX domain (Zhou et al., 2017). SNX proteins take part in protein sorting and transport across membranes by establishing protein-protein and protein-lipid interactions. Specifically, SNX10 interacts with V-ATPase and regulates its intracellular transport; accordingly, this autosomal recessive form of osteopetrosis results from altered transport of V-ATPase to the “corrugated edges” of osteoclasts and, consequently, their defective function. It has been suggested that SNX10 plays a role in the delivery and secretion of matrix metalloprotease 9, which is involved in the degradation of the extracellular matrix (Palagano et al., 2018).

Mutations in the SNX10 gene cause Västerbottenian osteopetrosis (named after the Swedish county), where the c.212+1G>T mutation in the SNX10 gene causing activation of a hidden splicing site in intron 4, leading to frameshift and stop codon formation (p.S66Nfs*15), occurs with a frequency of 1 : 93 in the population of this region. Genealogical studies and haplotype analysis have traced the origin of this mutation to a common ancestor in the early 19th century, and the age of the mutation is estimated to be approximately 950 years (Pangrazio et al., 2013; Stattin et al., 2017).

2 % of patients with ARO are deficient in the cytokine RANKL (receptor-activator nuclear kappa-B ligand) and 4.5 % are deficient in its receptor RANK. RANKL is encoded by the TNFSF11 gene, and binding to its receptor RANK, encoded by the TNFRSF11A gene, determines the activation of a downstream pathway that controls osteoclast differentiation and activation. The RANK/RANKL signaling pathway regulates the formation of mature osteoclasts from their precursors as well as their activity in bone remodeling. Disruption of this pathway results in a complete absence of mature osteoclasts in bone biopsy specimens. Patients with RANKL deficiency show severe osteopetrosis with slower disease progression compared to classical ARO (Penna et al., 2021).

Importantly, unlike TNFSF11 deficiency, osteopetrosis in patients with TNFRSF11A deficiency can be rescued by hematopoietic stem cell transplantation.

X-linked osteopetrosis is caused by mutations in the IKBKG gene. The IKBKG gene encodes NEMO, a regulatory subunit of the IKK complex (inhibitor of κB kinases) fundamental for the activation of the NF-κB (nuclear factor κB) transcription factor for the induction of osteoclastogenesis. NF-κB signal transduction involves a number of molecules (mainly kinases and transcription factors) that play a crucial role in the regulation of gene expression in many organs and in physiopathological conditions. In bone, it is supported by the fact that hypomorphic mutations in the IKBKG gene encoding a component of the IκB kinase complex required for inhibition of IκB-α and subsequent nuclear translocation of the released p65/p50 heterodimer are responsible for X-linked osteopetrosis with ectodermal dysplasia and immunodeficiency. These mutations are mainly localized in the zinc finger protein domain and lead to osteopetrosis by altering the RANKL/RANK signaling pathway (Frost et al., 2019; Jimi, Katagari, 2022).

Recently, a case was described in a newborn infant who died suddenly of unknown causes and pathological examination revealed a pathological increase in bone density associated with increased osteoblast function caused by de novo (c.1534_1535delinsAG (p.Asp512Ser)) mutation in the RELA gene (11q13.1). This mutation has been shown to disrupt NF-κB signaling in patient fibroblasts, which supports the hypothesis of possible changes in various vital functions (Frederiksen et al., 2016).

Severe combined immunodeficiency (SCID) is caused by a large deletion on chromosome 11 spanning the RAG1 and RAG2 genes and the 5′-region of TRAF6 (Weisz Hubshman et al., 2017).

Among the various adaptor molecules recruited via RANKL/RANK binding, TRAF6 (TNF receptor-associated factor 6) appears to be the most important. TRAF6 also acts downstream of the T- and B-cell receptor, leading to NF-κB activation

Several years ago, inactivation of the TRAF6 gene in mice was shown to cause severe osteopetrosis, and more recently, similar evidence was obtained in humans. In fact, a homozygous 2064 bp genomic deletion on chromosome 11 covering the 5′-region of the TRAF6, RAG1 and RAG2 genes (RAG proteins are necessary for B- and T-cell receptor recombination and for the survival and differentiation of these cells) was identified in two sibling patients with osteopetrosis and severe combined immune deficiency (SCID) by chromosomal microarray analysis. This genomic deletion covers the region above exon 1 and part of the non-coding sequences of exon 1. It is likely that these regions are regulatory; in fact, at the protein level, their deletion completely abolishes TRAF6 production. This mutation has been described in a single family, and the osteopetrosis was not generalized, but was pronounced in the pelvis and legs; because both patients, brother and sister, died at a very young age due to a severe immunological defect, it is currently difficult to predict the evolution of the disease in this particular case (Weisz Hubshman et al., 2017).

Mutations in the FERMT3 and CALDAGGEF1 genes cause osteopetrosis combined with leukocyte adhesion deficiency type III (LAD III).

The CALDAGGEF1 gene lies at the distal edge of the region of chromosome 11q13.1, is activated through diacylglycerol and Ca2+ binding, and is a guanine replacement factor for Rap1, a GTPase that plays an essential role in integrin activation. The gene encodes two proteins by alternative splicing, a cytosolic form of 68-kDa and a form of 72-kDa localized in the membrane through an additional amino-terminal myristoylated and palmitoylated domain (Svensson et al., 2009).

The FERMT3 gene (chromosome 11: 63.73–63.75 Mb) is located 0.5 Mb from CALDAGGEF1 on chromosome 11q13.1. The FERMT3 gene (representative of fermitin family 3) is expressed in hematopoietic cells and codes for kindlin-3, a member of the kindlin family that includes three different focal adhesion proteins involved in integrin activation. This process is necessary for cell adhesion, proliferation and migration, organization of the extracellular matrix, cell survival, proliferation, and differentiation.

Kindlin-3 is an intracellular protein bound to the actin cytoskeleton. It interacts with several classes of integrins and mediates their adhesive function and the transmission of signals from inside to outside, which is essential in bone for the resorptive activity of osteoclasts. Accordingly, kindlin-3 deficiency causes a major morphological change in osteoclasts and impairs their ability to attach to the bone surface. Mutations with a prematurely-terminating codon have been mainly described: nonsense mutations, splice defects, frameshifts, and, very rarely, missense mutations. Unfortunately, because the number of cases published in the literature is very limited, a gene-phenotypic correlation cannot be made at this time (Svensson et al., 2009).

Mutations in the LRRK1 (leucine-rich repeat kinase 1) gene are responsible for osteosclerotic metaphyseal dysplasia. The LRRK1 gene consists of 34 exons spanning about 150 bp on chromosome 15q26.3. LRRK1 encodes a multidomain protein of 2015 amino acids that contains ankyrin repeats, leucine-rich repeats), a C-terminal Roc (COR) domain and a serine-threonine kinase domain, and seven tryptophan-aspartic acid (WD) 40-domain dipeptides at the C-terminal

Mutations of the LRRK1 gene have been described in only five patients; a homozygous seven-nucleotide deletion in the last exon of the gene (c.5938_5944delGAGTGGT, p.Glu1980Alafs*66) was recently identified in one of these patients. This mutation is predicted to cause frameshift and premature termination with loss of the seventh tryptophanaspartic acid (WD) 40 domain. The WD40 domain, like other functional domains in the LRRK1 protein, mediates protein-protein interactions. In particular, it has been suggested that LRRK1 interacts with components of the c-Src signal transduction pathway to achieve cytoskeleton and “corrugated edge” rearrangement and podosome assembly. Accordingly, LRKK1-deficient osteoclasts are flat and large because they are unable to properly reorganize the cytoskeleton and resorb bone (Iida et al., 2016; Xing et al., 2017).

Another gene associated with osteopetrosis is MITF (microphthalmic-associated growth factor), which encodes a transcription factor that acts downstream of the RANK/ RANKL pathway. MITF deficiency is responsible for the COMMAD syndrome (Coloboma, Osteopetrosis, Microphthalmia, Macrocephaly, Albinism, and Deafness).

Microphthalmia-associated transcription factor (MITF) is a major helix-loop-helix-zipper transcription factor that forms homo/heterodimers that regulate gene expression in various tissues, so a range of phenotypes can reasonably be expected when it is mutated. In bone, MITF is thought to act along the RANKL/RANK signaling pathway downstream of NFATc1 to enhance NFATc1-dependent osteoclastogenic signaling.

Complex heterozygous mutations in the MITF gene have most recently been found in two unrelated patients with COMMAD syndrome manifesting coloboma, osteopetrosis, microphthalmia, macrocephaly, albinism and deafness. The identified mutations (c.952_954delAGA (p.Arg318del) and c.921G>C (p.Lys307Asn) in proband I; c.952A>G (p.Arg318Gly) and c.938-1G>A (p.Leu312fs*) in proband II) do not alter MITF dimerization, but rather its nuclear migration and DNA binding properties. This finding broadens the spectrum of phenotypes defined by MITF; in fact, unlike recessive mutations, dominant mutations are associated with Waardenburg type 2A syndrome and Titz syndrome, which share the characteristics of deafness and pigmentation deficiency. Overall, these data support an essential role of MITF in developmental processes as well as in cell differentiation and survival (George et al., 2016).

Poikiloderma with neutropenia is an autosomal recessive genodermatosis caused by mutations in the C16orf57 gene located on chromosome 16q21. To date, 17 mutations (deletions, nonsense mutations, and splice site mutations) have been identified in 31 patients with poikiloderma. The C16orf57 gene encoding phosphodiesterase is responsible for the modification and stabilization of small nuclear RNA U6 (USB1), which is an important element of the splicing mechanism (Colombo et al., 2012; Larizza et al., 2013).

Generalized osteopetrosis with severe cerebral malformation has been reported in consanguineous patients with mutations in the CSF1R gene who had osteopetrosis and cerebral malformations. The CSF1R gene encodes the M-CSF (macrophage colony-stimulating factor) receptor, which is a key transmembrane tyrosine kinase receptor that modulates microglial homeostasis, neurogenesis and neuronal survival in the CNS. CSF1R, which can be proteolytically cleaved into a soluble ectodomain and an intracellular protein fragment, supports myeloid cell survival when activated by two ligands, colony-stimulating factor 1 and interleukin 34 (Hu et al., 2021).

M-CSF is an important osteoclastogenic molecule, as well as RANKL, and it is well demonstrated in osteopetrotic mice with osteoclast deficiency lacking this cytokine. M-CSF receptor deficient mice (CSF1R) show a similar osteopetrotic phenotype; in addition, both models have defects in innate immunity, fertility and neurological function. Interestingly, dominant mutations in the CSF1R gene cause the adult form of encephalomyopathy, whereas just recently, a recessive mutation in this gene was thought to be responsible for the lethal complex phenotype in two siblings with generalized osteopetrosis and severe cerebral malformation. Exome sequencing in the blood parents of the deceased children revealed a heterozygous mutation (c.1620C>T (p.Tyr540*)) in the CSF1R gene that is predicted to result in a protein lacking the intracellular domain that is required for ligand-dependent dimerization and autophosphorylation. In the absence of a patient DNA sample, homozygosity for the CSF1R mutation has not been demonstrated in sick patients; therefore, these conclusions were not definitive. However, it would be interesting to analyze the gene in other patients with a similar phenotype trying to identify additional mutations as confirmation.

A rare form of osteopetrosis with low osteoclast content, called dysosteosclerosis, accompanied by red-purple macular atrophy, platyspondylitis, and metaphyseal osteosclerosis, is caused by mutations in the SLC29A3 gene (member 3 of the 29 solute carrier family), which codes for a highly expressed lysosomal nucleoside carrier in myeloid cells. The described mutations c.607T>C (p.Ser203Pro), c.1157G>A (p.Arg386Gln), c.1346C>G (p.Thr449Arg), c.303_320dup (p.102_107dup) identified in the SLC29A3 gene affect osteoclast function and differentiation, as suggested by reduced osteoclast numbers after in vitro differentiation from patient peripheral blood mononuclear cells and in patient bone biopsy samples (Palagano et al., 2018). More recently, a new splice site mutation in intron 6 of the TNFRSF11A gene has been described in one patient, indicating that TNFRSF11A is an additional gene responsible for dyosteosclerosis.

Osteopoikylosis, Buschke–Ollendorff syndrome, and melorheostosis are benign and more often asymptomatic conditions of osteopetrosis, diagnosed more often radiologically, and caused by mutations in the LEMD3 gene. LEMD3 is an integral protein of the inner nuclear membrane. It contains a nucleoplasmic N- and C-terminal domain and two helical transmembrane segments. The N-terminal segment shares a conserved globular domain of approximately 40 amino acids with other inner nuclear membrane proteins such as lamina-associated polypeptide 2 (LAP2) and emerin. The coding protein functions to counteract transforming growth factor-beta signaling at the inner nuclear membrane (Hellemans et al., 2004).

The gene responsible for pycnodysostosis is CTSK, located on chromosome 1 (1q21), encoding cathepsin K, a papain superfamily cysteine peptidase used by osteoclasts to degrade bone matrix and endowed with the unique ability to cleave collagen molecules in multiple sites. In addition, cathepsin K has recently been shown to cleave and activate matrix metalloproteinase 9 in vitro, indicating the presence of a protease signaling network likely significant in various physiopathological conditions. More recently, cathepsin K has been shown to contribute to the regulation of bone modeling by downregulating periostin, a cortical compartment matricellular protein required for Wnt-β-catenin-mediated periosteal formation (Pangrazio et al., 2014; Amr et al., 2021).

To date, about 60 different mutations have been described in the literature in patients of different geographic origins. Missense variants are the most frequent mutations; frameshifts, nonsense mutations, and splicing defects have also been identified. Mutations mainly occur in the mature CTSK protein, where exons 5 and 6 are “hot spots”. In addition, about 6 % of mutations are mapped to the pre-region, and 25 %, to the pro-region, which are short N-terminal domains necessary for proper protein localization, protein folding, and intracellular transport, respectively; the proregion is also necessary to keep the enzyme in an inactive state and is detached at low pH. However, genotype-phenotype correlations, which probably also explain atypical manifestations, have not been specifically investigated (Pangrazio et al., 2014).

Striated osteopathy with skull sclerosis is caused by mutations in the WTX gene (AMER1). This gene is located on chromosome Xq11.2 and contains 2 exons. The protein encoded by this gene enhances the activation of transcription by Wilms’ tumor protein and interacts with many other proteins. The prevalence of this form of osteopetrosis is 0.1:1,000,000 people (Jeoung et al., 2015). More than one hundred patients with this syndrome worldwide have been described, of which about one-third of the patients described are sporadic. Cranial sclerosis, in particular, is a clinically heterogeneous condition, ranging from mild skeletal manifestations to multisystem organ damage even within the same family.

## Conclusion

Osteopetrosis is a clinically and genetically heterogeneous group of disorders the diagnosis of which is complicated by the presence of different clinical forms and types of inheritance and the absence of a clear correlation between genotype and phenotype. Moreover, the mutations identified to date explain only 70 % of cases of osteopetrosis. The search for the molecular defects responsible for the remaining 30 % of the disease continues

The study of osteopetrosis is necessary for DNA diagnosis, treatment prescription, and prognosis. The study of osteopetrosis has shed light on little-known aspects of bone tissue cell biology and identified new mechanisms of osteoclast differentiation and function.

## Conflict of interest

The authors declare no conflict of interest.
